# Whole brain radiation therapy alone versus radiosurgery for patients with 1–10 brain metastases from small cell lung cancer (ENCEPHALON Trial): study protocol for a randomized controlled trial

**DOI:** 10.1186/s13063-018-2745-x

**Published:** 2018-07-16

**Authors:** Denise Bernhardt, Adriane Hommertgen, Daniela Schmitt, Rami El Shafie, Angela Paul, Laila König, Johanna Mair-Walther, Johannes Krisam, Christina Klose, Thomas Welzel, Juliane Hörner-Rieber, Jutta Kappes, Michael Thomas, Claus Peter Heußel, Martin Steins, Meinhard Kieser, Jürgen Debus, Stefan Rieken

**Affiliations:** 1 Department of Radiation Oncology, University Hospital Heidelberg, INF 400, 69120 Heidelberg, Germany; 2 Heidelberg Institute of Radiation Oncology (HIRO), Heidelberg, Germany; 3 Department of Thoracic Oncology. Translational Lung Research Centre Heidelberg (TLRC-H), Thoraxklinik, Heidelberg University, Heidelberg, Germany; 4 Department of Pneumology, Thoraxklinik, Heidelberg University, Heidelberg, Germany; 5 Clinical Cooperation Unit Radiation Oncology, German Cancer Research Center (DKFZ), Im Neuenheimer Feld 280, 69120 Heidelberg, Germany; 6 Heidelberg Ion-Beam Therapy Center (HIT), Im Neuenheimer Feld 450, 69120 Heidelberg, Germany; 7 Translational Lung Research Centre Heidelberg (TLRC-H), German Centre for Lung Research (DZL), Heidelberg, Germany; 8 Department of Neurooncology, University Hospital of Heidelberg, Im Neuenheimer Feld 672, 69120 Heidelberg, Germany; 9 Institute of Medical Biometry and Informatics, University of Heidelberg, Im Neuenheimer Feld 130.3, 69120 Heidelberg, Germany

**Keywords:** Small-cell lung cancer, Brain metastases, Stereotactic radiosurgery, Whole brain radiotherapy

## Abstract

**Background:**

Conventional whole brain radiotherapy (WBRT) has been established as the treatment standard in patients with cerebral metastases from small-cell lung cancer (SCLC), however, it has only modest efficacy and limited prospective data is available for WBRT as well as local treatments such as stereotactic radiosurgery (SRS).

**Methods/design:**

The present single-center prospective randomized study, conducted at Heidelberg University Hospital, compares neurocognitive function, as objectively measured by significant deterioration in Hopkins Verbal Learning Test – Revised total recall at 3 months. Fifty-six patients will be randomized to receive either SRS of all brain metastases (up to ten lesions) or WBRT. Secondary endpoints include intracranial progression (local tumor progression and number of new cerebral metastases), extracranial progression, overall survival, death due to brain metastases, local (neurological) progression-free survival, progression-free survival, changes in other cognitive performance measures, quality of life and toxicity.

**Discussion:**

Recent evidence suggests that SRS might be a promising treatment option for SCLC patients with brain metastases. The present trial is the first to prospectively investigate the treatment response, toxicity and neurocognition of WBRT and SRS in SCLC patients.

**Trial registration:**

Clinicaltrials.gov NCT03297788. Registered September 29, 2017.

**Electronic supplementary material:**

The online version of this article (10.1186/s13063-018-2745-x) contains supplementary material, which is available to authorized users.

## Background

Patients suffering from small cell lung cancer (SCLC) are at high risk for developing brain metastases (BM) during the course of their disease. Between 40% and 50% of patients develop BM until time of death [1] and the risk of developing BM further increases with prolonged survival [2]. Prophylactic cranial irradiation (PCI) is offered to limited disease patients if they respond to first line regime [3–5]. However, up to 10–15% of patients present with BM at initial diagnosis [6–8], and if magnetic resonance imaging (MRI) is used as a diagnostic tool for initial staging, the proportion increases to 15–20% [9]. Treatment options are usually limited to whole brain radiotherapy (WBRT) and palliative chemotherapy [10]. The actual effect of therapeutic WBRT has mainly been studied in small retrospective and non-randomized studies [2, 11–15]. Moreover, patients within recursive partitioning analysis (RPA) class III were commonly excluded from any prospective BM trials that involved WBRT [16, 17]. In a recent Japanese trial, prophylactic cranial irradiation did not result in longer overall survival compared with observation plus regular MRI follow-ups in patients with extensive disease (ED) SCLC [18]. PCI is therefore no longer recommended for patients with ED SCLC [19] when patients undergo regular MRI brain scans during follow-up [18]. Therefore, the number of patients with oligometastasic cerebral disease might rise.

The present trial aims to investigate the treatment response to ‘conventional whole brain radiotherapy’ and ‘stereotactic radiotherapy’ (SRS) in SCLC patients. The primarily investigated endpoint is neurocognitive function, as objectively measured by significant deterioration in Hopkins Verbal Learning Test – Revised (HVLT-R) total recall at 3 months [20]. Secondary endpoints include intracranial progression (local tumor progression, number of new cerebral metastases), extracranial progression, overall survival, death due to brain metastases, local (neurological) progression-free survival, progression-free survival, changes in other cognitive performance measures, quality of life, and toxicity.

## Methods/design

**Fig. 1:**
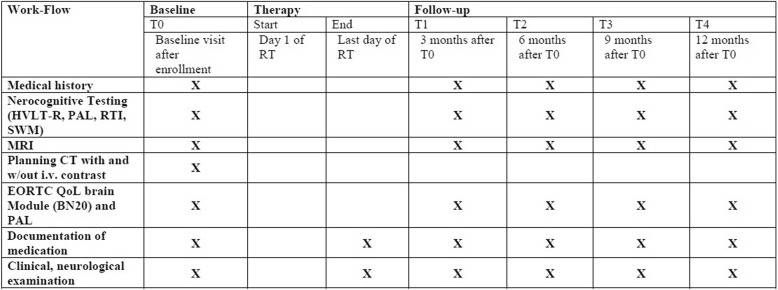
Intervention and assessment schedule for the ENCEPHALON trial

The study is a randomized phase II study with two study arms. The standard arm is WBRT and the experimental arm is SRS (Fig. 1). We hypothesized that patients treated with WBRT would have inferior neurocognitive function based on the HVLT-R [20] compared with patients treated with SRS alone.

### Recruitment and randomization

Eligible patients who present at the Departments of Radiation Oncology, University Hospital Heidelberg, Germany, will be recruited to the study. Eligibility requirements are:

### Inclusion criteria

- Histologically confirmed ED SCLC
- MRI-confirmed cerebral metastasis (not resected, maximum number of 10)
- Age ≥ 18 years of age
- For women with childbearing potential (and men), adequate contraception
- Ability of subject to understand character and individual consequences of the clinical trial
- Written informed consent (must be available before enrolment in the trial)

### Exclusion criteria

- Refusal of the patients to take part in the study
- Previous radiotherapy of the brain
- Patients who have not yet recovered from acute high-grade toxicities of prior therapies
- Known carcinoma < 5 years ago (excluding carcinoma in situ of the cervix, basal cell carcinoma, squamous cell carcinoma of the skin) requiring immediate treatment interfering with study therapy
- Pregnant or lactating women
- Participation in another clinical study or observation period of competing trials, respectively
- MRI contraindication (i.e., cardiac pacemaker, implanted defibrillator, certain cardiac valve replacements, certain metal implants)
- Karnofsky Performance Score < 60
- Simultaneous cytotoxic chemotherapy
- Last application of chemotherapy/immunotherapy/targeted therapy < 1 week before cerebral radiotherapy

After meeting eligibility criteria, 56 patients will be randomly assigned to SRS or WBRT. To achieve comparable intervention groups, patients will be allocated in a concealed fashion in a 1:1 ratio by means of randomization using a centralized web-based tool (www.randomizer.at). Randomization will be stratified with respect to time of appearance (synchronous vs. metachronous). Block randomization with varying block lengths will be performed to achieve equal group sizes in total. BM are defined as synchronous if discovered at the time of initial diagnosis of the primary tumor or within 3 months thereafter. All other patients will be classified as metachronous.

### Assessment of the primary and secondary endpoints

The primary endpoint is neurocognition after cerebral irradiation in SCLC patients treated with WBRT or SRS, defined as a drop of at least 5 points from baseline in HVLT-R total recall at 3 months. Secondary objectives are intracranial progression (local tumor progression, number of new cerebral metastases), extracranial progression, overall survival, death due to brain metastases, local progression-free survival, progression-free survival, changes in other cognitive performance measures, quality of life, and toxicity.

Time to progression is defined as the number of days from randomization to the first occurrence of the respective event. Overall survival time is defined as number of days from randomization until death or end of follow-up. For patients alive at the end of the study, the overall survival time will be censored at the time of the last visit or follow-up contact.

Time to death due to BM is defined as number of days from randomization until death due to BM or end of follow-up, where death due to BM is defined as death with intracranial progression as a component of cause of death. Locally progression-free survival time is defined as number of days from randomization until local tumor progression, death without prior local progression, or end of follow-up. The occurring events of interest will be classified as (1) progression of cerebral metastases present at baseline only, (2) occurrence of new cerebral metastases only, (3) simultaneous detection of progression of cerebral metastases present at baseline and of new metastases, and (4) death without local progression.

Time to extracranial progression is defined as number of days from randomization until extracranial progression or end of follow-up, where extracranial progression is the first date on which progressive disease outside the brain occurred according to the physician treating the primary disease. Progression-free survival time is defined as number of days from randomization until the first occurrence of intracranial or extracranial progression, death without prior progression, or end of follow-up. Intracranial progression is defined as occurrence of progressive disease concerning the pre-existing BM or the occurrence of new BM.

Local tumor progression (progressive disease, PD) is defined as occurrence of intracranial progression) in the area of the SRS.

Progression in the WBRT area is defined as occurrence of intracranial tumor progression (progression of existing lesions and/or occurrence of new lesions).

The European Organization for Research and Treatment of Cancer (EORTC) Questionnaire including brain module (BN20) and the EORTC Paired Associated Learning Questionnaire will be used. This study will use the International Common Terminology Criteria for Adverse Events (CTCAE) version 4.0 for toxicity and adverse event reporting.

Patients are followed within the trial protocol for 12 months after baseline visit (T0). After T0, patients are scheduled for follow-up visits every 3 months or as needed clinically, including contrast-enhanced MRI as well as thorough clinical-neurological assessment. Formal neurocognitive testing and quality of life instrument testing will be performed at baseline and during follow-up visits every 3 months. The computer-administered Cambridge Neuropsychological Test Automated Battery (CANTAB) will be used to examine specific components of cognition. The participants will be instructed to respond to stimuli presented on a computer screen by pressing a touch screen. Three tests were chosen to evaluate further neurocognitive changes: Paired Associated Learning for paired Associates Learning assesses visual memory and new learning [21]; Reaction Time to provide assessments of motor and mental response speeds [22], as well as measures of movement time, reaction time, response accuracy and impulsivity. Further, Spatial Working Memory will be used [22], which requires retention and manipulation of visuospatial information. Staff members administering the tests were trained and approved by the clinical neuropsychologist in the project group.

For the last patient in, the final study visit will be 12 months after baseline. This is considered the final study visit (last patient out). All other patients will be followed regularly as described in detail until death or until 12 months after baseline. Beyond that, all patients can be followed within routine clinical visits according to national guidelines and survival and progression data will be documented until ‘last patient out’. If death occurs at less than 12 months or patients leave the study prior to 12 months, they will be still included into the intention-to-treat population.

### Radiotherapy

#### Treatment planning for WBRT

For WBRT, patients will be immobilized using an individually manufactured head mask. For treatment planning, computed tomography (CT) without contrast, contrast-enhanced CT as well as MRI will be performed for optimal target definition. The target volume includes the whole brain. WBRT will be delivered by opposed lateral 6 MeV photon beams. Dose constraints of normal tissue will be respected according to QUANTEC reports [23, 24]. WBRT will be applied in 10 once-daily fractions each of 3 Gy, to a total dose of 30 Gy in 10 fractions to the whole brain.

#### Treatment planning for SRS

MRI and CT imaging are 3-dimensionally fused using a validated non-elastic imaging fusion algorithm and the fusion results are cross-checked by an experienced physician and adapted if necessary.

Organs at risk are contoured and adapted on the basis of CT scans and MRI. For target delineation, a gross target volume (GTV) and a planning target volume (PTV) are contoured. The basis for GTV definition is the contrast-based, T1-weighted, three-dimensional MPRAGE sequence. The GTV consists of all contrast-enhanced tissue associated with the target lesion and all additional tissue judged by an experienced physician to be part of the suspect target lesion (e.g. non-contrast-enhanced necrotic tissue within or adjoining cystic metastatic lesions). To the GTV, a PTV margin of 1 mm is added by isotropic expansion that can be slightly modified if deemed necessary by the treating physician (e.g., intersection with adjoining organs at risk).

Treatment planning for SRS will be performed using Accuray’s Multiplan or subsequent approved treatment planning systems for CyberKnife^®^.

SRS will be applied in one to a maximum of six fractions. For SRS, the dose prescription to the PTV will be as follows (risk adapted SRS dose prescription volume and location based):

- 20 Gy to the 70%-isodose (lesions < 2 cm max. diameter)
- 18 Gy to the 70%-isodose (lesions 2–3 cm max. diameter)
- 6 × 5 Gy to the conformally surrounding isodose (lesions > 3 cm max. diameter or brain stem)

Dose constraints of normal tissue will be respected according to QUANTEC reports [23, 24] and extensive clinical experience at our institution.

### Statistical analysis

The primary hypothesis of the trial is that there is a difference between the two treatment arms with respect to the primary endpoint, defined as a drop of at least 5 points from baseline in HVLT-R total recall at 3 months after baseline (T0). Chang et al. [25] observed deterioration probabilities of 0.64 for SRS + WBRT and 0.20 for SRS alone at 4 months after baseline. Based on those results, assuming a deterioration probability of 0.20 for the SRS arm and 0.64 for the WBRT arm in our trial, *n* = 19 patients per arm are required to demonstrate a difference between treatment arms applying a χ^2^ test at a two-sided significance level of α = 0.05 with a probability of 1–β = 0.8. Assuming exponentially distributed survival times with a median of 6 months for both groups, 29.3% of all randomized patients are expected to have died before the measurement of the primary endpoint. Thus, *n* = 28 patients per group are required to yield a sufficiently high power for a comparison of the deterioration rate within the two groups. Statistical analysis is based on the International Conference on Harmonization Guidelines Structure and Content of Clinical Study Reports and Statistical Principles for Clinical Trials. A detailed methodology for the statistical analysis will be described in the statistical analysis plan, which will be finalized before database lock. Statistical analysis will be performed using SAS v9.4 or higher.

### Efficacy evaluation

The primary hypothesis of the trial is that there is a difference between the two treatment arms with respect to the primary endpoint, defined as a drop of at least 5 points from baseline in HVLT-R total recall at 3 months. With π_SRS_ being the deterioration probability in the SRS arm and π_WBRT_ being the deterioration probability in the WBRT arm, the null hypothesis H_0_: π_SRS_ = π_WBRT_ is tested against its alternative H_1_: π_SRS_ ≠ π_WBRT_ at a two-sided significance level of α = 0.05 using a Cochran–Mantel–Haenszel test adjusting for the confounder time of appearance (synchronous vs. metachronous). Missing data for the primary outcome variable will be replaced by using multiple imputation which takes the covariates of treatment group, time of appearance (synchronous vs. metachronous), and the baseline HVLT-R total recall score into account by application of the fully conditional specification method [26]. For the secondary time-to-event endpoints overall survival, local progression-free survival, and locoregional progression-free survival, median event times and 1-year rates will be given with 95% confidence intervals and Kaplan–Meier curves will be calculated for both treatment groups. A (descriptive) log-rank test stratified for time of appearance (synchronous vs. metachronous) will be performed in order to assess differences between the two treatment groups, and a descriptive *P* value will be given. A Cox proportional hazard model with overall survival as dependent variables well as treatment group and time of appearance as independent factors will be fitted to estimate the hazard ratio for the treatment group together with a 95% confidence interval.

All further secondary outcomes will be analyzed descriptively, and descriptive *P* values will be reported together with corresponding 95% confidence intervals.

### Ethical issues, information, and safety

The study protocol, Patient Information sheet, and Declaration of Informed Consent was approved by the Heidelberg University Ethics Committee (S-470/2017). The procedures described in the submitted study protocol regarding the performance, evaluation, and documentation of this study has been selected in such a way that the principles of the Good Clinical Practice (GCP) guidelines are observed. The regulations regarding medical confidentiality and data protection are fulfilled. Informed consent will be obtained from all participants in the study.

Concerning radiation protection law (StrSchV), the authors of this protocol presume that a submission to the Bundesamt für Strahlenschutz (BfS) is not required. To confirm this position, the investigators submitted this protocol to the expert commission of the German Society of Radiation Oncology ENCEPHALON Clinical Trial Protocol Version 1.0, Date 07/2017 (DEGRO No. 132) (Additional file 1; SPIRIT Checklist).

## Discussion

The primary aim of this trial is to exploratively investigate the effect of SRS compared to WBRT in patients with BM from SCLC. Currently, according to national guidelines, the recommendation for patients with BM from SCLC, regardless of the number of BM, is WBRT [10]. The actual evidence behind those recommendations is low and mainly based on retrospective studies from the last three decades and is analogically reasoned by previous PCI studies [3–6, 27]. In a recent Japanese trial, prophylactic cranial irradiation did not result in longer overall survival compared with observation in patients with ED SCLC and PCI is therefore no longer recommended for patients with extensive disease SCLC when patients receive regular MRI examinations during follow-up. Furthermore, if SCLC patients are regularly checked with MRI, the actual number of patients with limited number of BM might rise. The general use of WBRT in SCLC patients is additionally supported by the general paradigm of a diffuse intracranial disease pattern; even so, these beliefs derive from a pre-MRI era. The EORTC conducted a prospective, phase II study between 1989 and 1995 that included patients (*n* = 22) with brain-only metastases SCLC to evaluate the efficacy of WBRT [28]. The median response duration in patients with an objective response was 5.4 months, and the median survival of all patients was 4.7 months. A number of retrospective studies investigated prognostic factors and identified subgroups of SCLC patients with a favorable prognosis [15, 29–31]. In a recent report [5], we investigated 229 SCLC patients with BM from SCLC; median overall survival after WBRT was 6 months. The main prognostic factors associated with overall survival were performance status, time of appearance of intracranial disease (synchronous vs. metachronous), initial response to chemotherapy, and higher RPA class. Interestingly, patients in RPA class I showed a median survival after WBRT of 17 months and had a comparable outcome to patients with non-cerebral disease treated with PCI. Furthermore, 39% of patients had 1–5 BM and the majority of patients received staging with cerebral MRI prior to treatment [32]. The recently developed disease-specific prognostic score for patients with BM from SCLC was even more prognostic than RPA score and diagnosis-specific graded prognostic assessment score [29] and revealed a subgroup of patients with a very short survival (class I) and a subgroup with a favorable prognosis (class II) [33]. The disease-specific prognostic score for patients with BM from SCLC will be validated within the ENCEPHALON trial. Because of the multiple prognostic factors and differences in outcomes in patients with BM from SCLC, a one- fits-all treatment framework, in which all patients are automatically recommended for WBRT, is no longer appropriate and the actual evidence for this recommendation is missing.

Radiosurgery and surgery are possible treatment options in patients with a limited number of BM and for patients with BM of less than 3 cm in diameter from solid tumors, except SCLC patients. For patients with 2–4 metastases and with a life expectancy of more than 3 months, radiosurgery should be used rather than WBRT [34–38]. A recent Japanese trial investigated the effect of SRS in 1194 patients with multiple brain metastases from solid tumors. The authors concluded that SRS in patients with 5–10 BM is non-inferior to that in patients with 2–4 BM. Considering the minimal invasiveness of stereotactic radiosurgery and the fewer side effects than with WBRT, SRS might be a suitable alternative for patients with up to 10 brain metastases [39].

For SCLC patients there is only limited prospective data available regarding locally ablative treatments or SRS. However, a number of retrospective studies investigated the effect of SRS as a treatment in a primary setting and as a salvage option after prior WBRT or PCI in SCLC patients [32, 40–44]. Patients who received SRS instead of WBRT showed a comparable or even better survival compared to patients with WBRT. This is, of course, biased by the retrospective design and the limited number of BM in the patient group that received SRS. On the other hand, this implies that a subgroup of patients might be suitable for SRS. In a prospective cohort trial, Li et al. [45] evaluated SRS versus SRS + WBRT and WBRT alone in patients with a single BM of SCLC or non-SCLC. The study did not reveal a statistically significant difference concerning median survival (9.3 vs. 10.6 months) or recurrence and progression. The authors concluded that SRS-alone and SRS + WBRT seem superior to WBRT-alone in prolonging overall survival, local control, and improving quality of life in patients with single BM from lung carcinoma.

Chang et al. [25] prospectively evaluated neurocognitive outcome in 58 patients with 1–3 BM from solid tumors and were randomly assigned to SRS + WBRT or SRS alone. The primary endpoint was neurocognitive function measured as a significant deterioration (5-point drop compared with baseline) in HVLT-R total recall at 4 months. After 58 patients were recruited, the trial was stopped on the basis that there was a high probability (96%) that patients receiving SRS plus WBRT were significantly more likely to show a decline in learning and memory function at 4 months than patients assigned to receive SRS alone. SCLC patients often die from thoracic progression, rather than of progression of BM. Therefore, especially for a patient group with a favorable prognosis, the preservation of cognitive function and quality of life is essential.

### Trial status

Recruiting.

## Additional file

Additional file 1: SPIRIT 2013 Checklist: Recommended items to address in a clinical trial protocol and related documents*. (PDF 205 kb)

## Abbreviations

BM: brain metastases
CT: computed tomography
ED: extensive disease
EORTC: European Organization for Research and Treatment of Cancer
GTV: gross target volume
HVLT-R: Hopkins Verbal Learning Test – Revised
MRI: magnetic resonance imaging
PCI: prophylactic cranial irradiation
PTV: planning target volume
RPA: recursive partitioning analysis
SCLC: small cell lung cancer
SRS: stereotactic radiotherapy
WBRT: whole brain radiotherapy
