# Supplementary material for: Whole brain radiation therapy alone versus radiosurgery for patients with 1–10 brain metastases from small cell lung cancer (ENCEPHALON Trial): study protocol for a randomized controlled trial
Source: Trials. 2018 Jul 16;19:388. doi: 10.1186/s13063-018-2745-x (PMC6048892; doi:10.1186/s13063-018-2745-x)
Supplement: Supplementary file 1 — SPIRIT 2013 Checklist: Recommended items to address in a clinical trial protocol and related documents*. (PDF 205 kb) [file 13063_2018_2745_MOESM1_ESM.pdf]

SPIRIT 2013 Checklist: Recommended items to address in a clinical trial protocol and related documents*

Section/item	Item No	Description	Addressed on page number
**Administrative information**			
Title	1	**Whole brain radiation therapy (WBRT) alone versus radiosurgery (SRS) for patients with 1-10 brain metastases from small cell lung cancer (ENCEPHALON Trial)**	______________
Trial registration	2a	Trial registration: Clinicaltrials.gov NCT03297788. Registered September 29, 2017	______________
	2b	N/A	______________
Protocol version	3	V2.0	______________
Funding	4	Research grant financed by Accuray Inc., Sunnyvale California. The funding source has no role in study design, data collection, data analysis, data interpretation, or writing of the report.	______________

Roles and responsibilities 5a

Denise Bernhardt[1,2], Adriane Hommertgen[1,2], Daniela Schmitt[1,2], Rami El Shafie[1,2], Angela Paul[1,2], Laila König[1,2], Johanna Mair-Walther[8], Johannes Krisam[9], Christina Klose[9], Thomas Welzel[1,2], Juliane Hörner-Rieber[1,2], Jutta Kappes[4]; Michael Thomas[3,7], Claus Peter Heußel[7,8,9], Martin Steins[3,7], Meinhard Kieser[9] Jürgen Debus[1,2,5,6], Stefan Rieken[1,2]

1. University Hospital Heidelberg, Department of Radiation Oncology, INF 400, 69120 Heidelberg, Germany
2. Heidelberg Institute of Radiation Oncology (HIRO), Germany
3. Department of Thoracic Oncology, Thoraxklinik, Heidelberg University, Heidelberg, Germany Translational Lung Research Centre Heidelberg (TLRC-H),
4. Department of Pneumology, Thoraxklinik, Heidelberg University, Heidelberg, Germany
5. Clinical Cooperation Unit Radiation Oncology, German Cancer Research Center (DKFZ), Im Neuenheimer Feld 280, 69120 Heidelberg, Germany
6. Heidelberg Ion-Beam Therapy Center (HIT), Im Neuenheimer Feld 450, 69120 Heidelberg, Germany
7. Translational Lung Research Centre Heidelberg (TLRC-H), German Centre for Lung Research (DZL)
8. University Hospital of Heidelberg, Department of Neurooncology, Im Neuenheimer Feld 672, 69120 Heidelberg, Germany
9. Institute of Medical Biometry and Informatics, University of Heidelberg, Im Neuenheimer Feld 130.3, 69120 Heidelberg, Germany

D. Bernhardt developed and planned this trial under the supervision of J Debus, M. Thomas, M. Steins and S. Rieken, who is the principal investigator. D. Bernhardt, R. El Shafie, J. Hoerner-Rieber, A. Paul, J. Kappes and L. König perform patient treatment and clinical assessments. T. Welzel and C.P. Heussel are the supervising diagnostic radiologists. D. Schmitt is responsible for treatment planning, dosimetry and plan verification. J. Krisam, and M. Kieser are the trial statisticians and responsible for statistical planning and statistical analysis. C. Klose is responsible for data management. J. Mair-Walther is responsible for neurocognitive testing. A. Hommertgen coordinates the study. All authors read and approved the final manuscript.

5b Name and contact information for the trial sponsor
Irmtraud Gürkan

Financial Director Universitätsklinikums Heidelberg

Im Neuenheimer Feld 400, 69120 Heidelberg, germany

Phone : 06221-56-7002 Phone: 06221 / 56-8202

Fax : 06221-56-4888 Fax: 06221 / 56-5353

e-mail: irmtraud.guerkan@med.uniheidelberg.De

5c The funding source has no role in study design, data collection, data analysis, data interpretation, or writing of the report.

5d NA

Introduction

Background and rationale 6a

A. 1.1 Scientific Background

Patients suffering from BM from SCLC have a poor prognosis with a median survival ranging between 2-14 months (Quan et al. 2004; Bernhardt 2017). Treatment options for BM in SCLC are usually limited to WBRT, steroids or palliative chemotherapy. SCLC patients demonstrate an exception in the treatment of BM, because treatment options for a limited number of BM from other solid tumors commonly include surgery or SRS with or without WBRT. Even though SCLC is a radiosensitive tumor, higher doses are commonly not applied (Fertil & Malaise 1985). Locally ablative treatments like SRS or surgery are less frequently used in patients with BM from SCLC as compared to other types of cancer due to the high incidence of brain metastases in SCLC and the increased likelihood of a diffuse failure pattern (Komaki et al. 1981; Carmichael et al. 1988). It is of general belief that BM from SCLC are rarely solitary and usually occur at multiple sites (Postmus 1995b; Newman & Hansen 1974). We could not confirm these findings from this analysis as we found 1-5 BM in 39 % of our patients (Bernhardt 2017).

WBRT, with a treatment time of about two weeks, is commonly the technique of choice for SCLC patients with any number of BM. The SCLC-specific general assumption of diffuse intracranial tumor cell spread and high risk of diffuse failure patterns has prevented radiation oncologists from adopting locally ablative therapies as a treatment in this cohort. Besides, in other solid tumors the risk for intracranial failure is also higher if SRS is used alone compared to WBRT plus SRS. Nevertheless, a higher intracranial progression rate did not impact OS as seen in a previous analysis by Aoyama et al. (Aoyama et al. 2006). In a recent Japanese trial, prophylactic cranial irradiation did not result in longer overall survival compared with observation in patients with ED SCLC. PCI is therefore no longer recommended for patients with ED SCLC when patients receive regularly MRI examinations during follow-up (Takahashi et al. 2017). Therefore, the number of patients with oligometastasic cerebral disease might rise.

In previous retrospective analyses, several prognostic factors for patients with BM have been identified. Significant negative prognostic factors were lower KPS, higher age, presence of extracranial metastases, and number of BMs (Sperduto et al. 2010; Sperduto et al. 2012). Due to the limited patient numbers in previous analysis, SCLC patients have been grouped with other solid tumors, especially NSCLC or the number of patients was even too low to reasonably asses valuable prognostic factors (Gaspar et al. 1997; Schwer & Gaspar 2006). In our in house study, the main prognostic factor has been performance status, response to initial chemotherapy, and time of appearance (synchronous vs. metachronous) (Bernhardt

	6b	There exists strong and prospective data supporting the hypothesis that SRS for up to ten cerebral metastases is non-inferior to WBRT, the current standard of care for those patients, that therapy-associated toxicity can even be reduced by SRS and that local tumor control can be improved. Those findings have already been incorporated within the respective NCCN guidelines (Yamamoto et al. 2014; Bower & Waxman 2011). This data is based on SRS performed with Leksell Gamma Knife, providing optimum precision and dose conformity to achieve a minimum of toxicity. Accuray's Cyberknife, with which treatment in this study will be performed, has been established to compare favorably to Gamma Knife in terms of precision and dose conformity (Chang et al. 2003).	
Objectives	7	The purpose of this trial is to explore the neurocognitive response in patients with brain metastases from SCLC treated with WBRT or SRS. We proposed that patients treated with SRS would have inferior neurocognitive function based on the Hopkins Verbal Learning Test–Revised (HVLT–R) compared with patients treated with SRS alone.	
Trial design	8	The primary objective is the exploratory investigation of neurocognition after cerebral irradiation in SCLC patients treated with WBRT or SRS, defined as a drop of at least 5 points from baseline in HVLT-R total recall at 3 months after baseline. After initiation of the study, patients will consecutively be screened and eligible patients will be enrolled into the study. To achieve comparable intervention groups, patients will be allocated in a concealed fashion in a 1:1 ratio by means of randomisation using a centralised web-based tool (www.randomizer.at). Randomization will be stratified with respect to time of appearance (synchronous vs. metachronous). Block randomisation with varying block lengths will be performed to achieve in total equal group sizes. Patients will be randomized to SRS or WBRT.	

Methods: Participants, interventions, and outcomes

Study setting	9	Single-Centre, randomized trial: The Department of Radiation Oncology treats patients with SCLC within the National Center of Tumor Diseases (NCT) and the Thoraxklinik Heidelberg. In this context, patients with primary and secondary brain tumors are treated in the interdisciplinary setting consisting of pulmonologist, oncologists, neurosurgeons, neurooncologist, neuroradiologist as well as radiation oncologist. Therefore, patients will be provided the best possible oncological care on a professional base. The center has experience and expertise in high-precision radiation treatments, and SRS for brain metastases is established as a standard treatment approach for certain patients with brain metastases (Klaus K Herfarth et al. 2003). At the Department of Radiation Oncology at Heidelberg University Hospital, SRS has been performed for almost three decades for a variety of indications and considerable expertise in that field has been established (Klaus K. Herfarth et al. 2003).	______________

Eligibility criteria 10

C. Inclusion Criteria

- histologically confirmed small cell lung cancer
- MR-imaging confirmed cerebral metastasis (not resected, maximum number of 10)
- age ≥ 18 years of age
- For women with childbearing potential, (and men) adequate contraception.
- Ability of subject to understand character and individual consequences of the clinical trial
- Written informed consent (must be available before enrolment in the trial)

D. Exclusion Criteria

- refusal of the patients to take part in the study
- previous radiotherapy of the brain
- Patients who have not yet recovered from acute high-grade toxicities of prior therapies
- Known carcinoma < 5 years ago (excluding Carcinoma in situ of the cervix, basal cell carcinoma, squamous cell carcinoma of the skin) requiring immediate treatment interfering with study therapy
- Pregnant or lactating women
- Participation in another competing clinical study or observation period of competing trials, respectively
- MRI contraindication (i.e. cardiac pacemaker, implanted defibrillator, certain cardiac valve replacements, certain metal implants)
- KPS <60
- Simultaneous cytotoxic chemotherapy
- Last application of chemotherapy/immunotherapy/targeted therapy <1 week before cerebral radiotherapy

Radiation Therapy

Treatment Planning for WBRT

For WBRT, patients will be immobilized using an individually manufactured head mask. For treatment planning, CT without contrast, contrast-enhanced CT as well as MR-imaging will be performed for optimal target definition. For treatment planning, CT without contrast as a virtual simulation will be performed. TV includes the whole brain. WBRT will be delivered by opposed lateral 6 MeV photon beams.

Dose constraints of normal tissue will be respected according to QUANTEC reports (Bentzen et al. 2010; Marks et al. 2010).

Treatment Planning for SRS

MRI and CT imaging are 3-dimensionally fused using a validated non-elastic imaging fusion algorithm and the fusion results are cross-checked by an experienced physician and adapted if necessary.

Organs at risk (OAR) are contoured and adapted on the basis of CT scan and MRI. For target delineation a GTV and a PTV are contoured. The basis for GTV definition is the contrast-based T1-weighted three-dimensional MPRAGE sequence. The GTV consists of all contrasted tissue associated with the target lesion and all additional tissue judged by an experienced physician to be part of the suspect target lesion (e.g. not contrasted necrotic tissue within or adjoining cystic metastatic lesions). To the GTV a PTV margin of 1 mm is added by isotropic expansion that can be slightly modified if deemed necessary by the treating physician (e.g. intersection with adjoining OAR).

Treatment planning for SRS will be performed using Accuray's Multiplan or subsequent approved treatment planning systems for Cyberknife.

Dose Prescription

WBRT will be applied in 10 fractions with single doses of 3 Gy to the whole brain.

For SRS the dose prescription to the PTV will be as follows:

- 20 Gy to the 70%-isodose (lesions < 2 cm max. diameter)
- 18 Gy to the 70%-isodose (lesions 2 - 3 cm max. diameter)
- 6 x 5 Gy to the conformally surrounding isodose (lesions > [illegible] cm max. diameter)

11b

Withdrawal of Patients

A subject may voluntarily discontinue participation in this study at any time at their own request or at request of the legal representative. In addition, study treatment will be discontinued if unmanageable toxicity is documented, or if the Sponsor or Principal Investigator makes a decision to terminate the study. A subject will be withdrawn from the protocol if, in the investigator’s opinion, continuation of the trial would be detrimental to the subject’s well-being. If the subject withdraws from the trial and also withdraws consent for disclosure of future information, no further evaluations should be performed, and no additional data should be collected. The sponsor may retain and continue to use any data collected before such withdrawal of consent.

Handling of Withdrawals

In all cases, the reason for withdrawal must be recorded in the CRF and in the subject’s medical records. In case of withdrawal of a subject at his/ her own request, the reason can be asked and documented. All efforts will be made to follow up the subjects and, all examinations scheduled for the final trial day will be performed as far as possible on all patients and documented. All ongoing Adverse Events (AEs)/ Serious Adverse Events (SAEs) of withdrawn patients have to be followed up until no more signs and symptoms are verifiable or the subject is on stable condition.

Replacement of Patients

Patients will not be replaced if consent is withdrawn retrospectively.

11c NA

11d No Simultaneous Chemotherapy or immunotherapy

Outcomes	12	Intracranial progression (local tumour progression, number of new cerebral metastases)<br>Extracranial progression<br>Overall survival<br>Death due to brain metastases<br>Locally progression-free survival<br>Progression-free survival<br>Changes in other cognitive performance measures<br>Quality of life<br>Toxicity	
Participant timeline	13	View end of document	

Sample size	14	56 patients<br>The primary hypothesis of the trial is that there is a difference between the two treatment arms with respect to the primary endpoint, defined as a drop of at least 5 points from baseline in HVLT-R total recall at 3 months after baseline. Chang et al. 2009 observed deterioration probabilities of 0.64 for SRS+WBRT and 0.20 for SRS alone at 4 months after baseline (Chang et al. 2009). It is assumed that deterioration rates at 3 months after baseline and 4 months after baseline are comparable. Based on those results, assuming a deterioration probability of 0.20 for the SRS arm and 0.64 for the WBRT arm in our trial, n=19 patients per arm are required to demonstrate a difference between treatment arms applying a chi-squared test at a two-sided significance level of α=0.05 with a probability of 1-β=0.8. Assuming exponentially distributed survival times with a median of 6 months after baseline for both groups, 29.3% of all randomized patients are expected to have died before the measurement of the primary endpoint. Hence, n=28 patients per group are required to yield a sufficiently high power for comparison of the deterioration rate within the two groups. The problem of missing values for the primary outcome will be partially handled by using a predefined imputation strategy (see Section 10.4 on statistical methods). It is assumed that the use of a Cochran–Mantel–Haenszel test taking the covariate time of appearance (synchronous vs. metachronous) into account will yield an increase in power. Sample size calculation was carried out using the statistical software ADDPLAN v6.1.	______________
Recruitment	15	High volume medical centre; National cancer centre	______________

Methods: Assignment of interventions (for controlled trials)

Allocation:

Sequence generation	16a - 17b	After initiation of the study, patients will consecutively be screened and eligible patients will be enrolled into the study. To achieve comparable intervention groups, patients will be allocated in a concealed fashion in a 1:1 ratio by means of randomisation using a centralised web-based tool (www.randomizer.at). Randomization will be stratified with respect to time of appearance (synchronous vs. metachronous). Block randomisation with varying block lengths will be performed to achieve in total equal group sizes.	______________

Methods: Data collection, management, and analysis

Data collection methods	18a/b	For this trial, relevant data will be documented in electronic CRFs. All findings including clinical and laboratory data will be documented by the investigator or an authorized member of the study team in the subject's medical record and in the eCRF. The investigator is responsible for ensuring that all sections of the CRF are completed correctly and that entries can be verified against source data. In some cases, the CRF, or part of the CRF, may also serve as source documents: Karnofsky Performance Status, Documentation of Clinical-Neurological Examination. In these cases, a document should be available at the investigator's site and clearly identify those data that will be recorded in the CRF, and for which the CRF will stand as the source document.	______________

Data management	19	The Institute of Medical Biometry and Informatics (IMBI) is in charge of the data management within the trial. An electronic case report form (eCRF) will be used for data collection. Any entry and correction in the remote data entry system will be documented automatically in an audit file. Completeness, validity and plausibility of data will be checked in time of data entry (edit-checks) and using validating programs, which will generate queries. The investigator or the designated representatives are obliged to clarify or explain the queries. If no further corrections are to be made in the database, it will be closed and used for statistical analysis. All data management procedures will be carried out on validated systems and according to the current Standard Operating Procedures (SOP) of the IMBI that guarantee an efficient conduct which is in compliance with GCP. At the end of the study, the data will be transformed into different data formats (e.g. csv-files) to ensure that it will be possible to reuse the data.<br>According to the §13 of the German GCP-Regulation all important trial documents (e.g. CRF) will be archived for at least 10 years after the trial termination. According to the §28c of the German X-Ray Regulation (RöV) and the §87 of the German Radiation Protection Regulation (StrlSchV) the informed consent forms including patients' consent for trial participation, application of irradiation and data transmission to the competent authority will be archived for at least 30 years after the trial termination.<br>The Study Center at the Department of Radiation Oncology will be responsible for archiving TMF including protocol, CRFs, report etc.	

Statistical methods	20a-c	Statistical analysis is based on the International Conference on Harmonization (ICH) Guidelines “Structure and Content of Clinical Study Reports” and “Statistical Principles for Clinical Trials”.<br>All data recorded in the CRF describing the sample, the efficacy and the safety will be analyzed descriptively. Categorical data will be presented in contingency tables with frequencies and percentages. Continuous data will be summarized with at least the following: sample size, median, quartiles, mean, standard deviation, minimum and maximum.<br>A detailed methodology for the statistical analysis will be described in the statistical analysis plan (SAP), which will be finalized before data base lock. Statistical analysis will be performed using SAS v9.4 or higher. Descriptive statistics will be performed for all demographic data and baseline characteristics by tabulating absolute and relative frequencies for categorical and mean, standard deviation, median, inter-quartile range, minimum and maximum for continuous variables.	______________

Methods: Monitoring

Data monitoring	21a	The investigator(s) will archive all trial data (source data and Investigator Site File (ISF) including subject identification list and relevant correspondence) according to the section 4.9 of the ICH Consolidated Guideline on GCP (E6) and to local law or regulations. The Subject Identification List will be archived for at least 15 years after the trial termination. If an investigator relocates, retires, or for any reason withdraws from the study, the principal investigator should be notified prospectively. The study records must be transferred to an acceptable designee, such as another investigator or another institution. To assure the safety of the patients, the PI and study coordinator closely monitors the course of the trial, especially with respect to the safety-relevant results and a potential recommendation to the principal investigator to stop the study early.	______________
	21b	No interim analyses	______________

Harms 22 A pre-existing disease or symptom will not be considered an adverse event unless there will be an untoward change in its intensity, frequency or quality. This change will be documented by an investigator. Each AE developing during study treatment or within 30 days after completion of study treatment (RT).. The investigator is responsible to perform and consider all required therapeutic measures and methods to follow-up this condition. An event, which occurs in conjunction or association with tumor progression will not be considered an AE or subsequently as an SAE unless more severe than expected.

Any SAE that occurs outside the SAE detection period (after the 30 day period) considered to be reasonably related to the investigational treatment or study participation have to be documented and reported. This must be done within 24 hours of the initial observation of the event. The principal investigator will decide if these events are related to the protocol treatment (i.e. unrelated, likely related, and not assessable) and the decision will be recorded on the Serious Adverse Event form, if necessary with the reasoning of the principal investigator.

The investigator is obligated to assess the relationship between investigational treatment and the occurrence of each AE/SAE. A "reasonable possibility" is meant to convey that there are facts/evidence or arguments to suggest a causal relationship, rather than a relationship cannot be ruled out. The investigator will use clinical judgement to determine the relationships. Alternative causes, such as natural history of the underlying diseases, concomitant therapy, other risk factors, and the temporal relationship of the event to the investigational treatment will be considered and investigated. Pursuant to the GCP Regulation, the EC will be informed of all suspected serious unexpected adverse reactions (SUSARs) and all AEs resulting in death or being live threatening occurring during the trial. The EC will be informed in case the risk/ benefit assessment did change or any others new and significant hazards for patients' safety or welfare did occur. Furthermore, a report on all observed serious adverse events (SAEs) will be submitted once a year – Annual Safety Report. The EC must be informed of the end of the trial. They will be provided with a summary of trial results within one year after the end of clinical phase (LSO).

Auditing	23	Regulatory authorities and an auditor authorized by the sponsor may request access to all source documents, CRF, and other trial documentation. Direct access to these documents must be guaranteed by the investigator who must provide support at all times for these activities.	______________

Ethics and dissemination

Research ethics approval	24	The local regulatory authorities and responsible for each particular investigator the competent higher federal authority will be informed, as required, before the beginning, during and at the end of the trial according to the applicable regulations. In Germany, a favorable opinion from the ethics committee must be obtained before a clinical trial is started. Concerning radiation protection law (StrSchV), the authors of this protocol presume that a submission to the Bundesamt für Strahlenschutz (BfS) is not required. This assumption is based on the fact that all treatments within this protocol are clinically indicated and performed within the medical responsibility of the participating centres. To confirm this position, the investigators submitted this protocol to the expert commission of the German Society of Radiation Oncology ENCEPHALON Clinical Trial Protocol Version 1.0, Date 07/2017 (DEGRO Anfrage 132). DEGRO, which has been established in cooperation with the BfS to judge the necessity of a BfS-submission of study protocols regarding radiotherapy. The expert commission of the DEGRO decided that a submission to the BfS is not required. A copy of the decision letter is attached. Furthermore, local ethics committee positively evaluated the study.	______________
Protocol amendments	25	NA	______________
Consent or assent	26a	Local Physiciac	______________
	26b	NA	______________

Confidentiality	27	According to the §13 of the German GCP-Regulation all important trial documents (e.g. CRF) will be archived for at least 10 years after the trial termination. According to the §28c of the German X-Ray Regulation (RöV) and the §87 of the German Radiation Protection Regulation (StrlSchV) the informed consent forms including patients' consent for trial participation, application of irradiation and data transmission to the competent authority will be archived for at least 30 years after the trial termination.<br>The Study Center at the Department of Radiation Oncology will be responsible for archiving TMF including protocol, CRFs, report etc.<br>The investigator(s) will archive all trial data (source data and Investigator Site File (ISF) including subject identification list and relevant correspondence) according to the section 4.9 of the ICH Consolidated Guideline on GCP (E6) and to local law or regulations. The Subject Identification List will be archived for at least 15 years after the trial termination. If an investigator relocates, retires, or for any reason withdraws from the study, the principal investigator should be notified prospectively. The study records must be transferred to an acceptable designee, such as another investigator or another institution.	______________
Declaration of interests	28	The trial is financed using funds of the Department of Radiation Oncology at the University Hospital of Heidelberg.<br><br>*The trial is financially supported by Accuray International 9 Avenue de la Longeraie, CH-1110 Morges, Switzerland14.2 Financial Disclosure*<br><br>According to the research agreement of our clinic with Accuray Medical Systems, this trial is financially supported. Accuray Medical Systems is not involved in the design of the study, and collection/storage/analysis of the data gathered in this study.	______________

Access to data	29	All data will be entered in a study specific database as recorded in the eCRF. All missing data or inconsistencies will be reported back to the investigators and clarified by the responsible investigator. If no further corrections are to be made in the database it will be declared closed and used for statistical analysis. All investigators have access.	______________
Ancillary and post-trial care	30	NA	______________
Dissemination policy	31a	NA	______________
	31b	All information concerning the trial is confidential before publication. Publication will be prepared under the lead of the principal investigator of the study. The first and last authorship are reserved for the principal investigator and the study coordinator of the study if both do not wish to transfer their authorship to a third person. All data will be published independently of the results of the trial.	______________
	31c	NA	______________
**Appendices**			
Informed consent materials	32	Model consent form and other related documentation given to participants and authorised surrogates	______________
Biological specimens	33	NA	______________

*It is strongly recommended that this checklist be read in conjunction with the SPIRIT 2013 Explanation & Elaboration for important clarification on the items. Amendments to the protocol should be tracked and dated. The SPIRIT checklist is copyrighted by the SPIRIT Group under the Creative Commons "Attribution-NonCommercial-NoDerivs 3.0 Unported" license.

Work-Flow	Baseline	Therapy		Follow-up			
	T0	Start	End	T1	T2	T3	T4
	Baseline visit after enrollment	Day 1 of RT	Last day of RT	3 months after T0	6months after T0	9 months after T0	12 months after T0
**Medical history**	**X**			**X**	**X**	**X**	**X**
**Nerocognitive Testing (HVLT-R, PAL, RTI, SWM)**	**X**			**X**	**X**	**X**	**X**
**MRI**	**X**			**X**	**X**	**X**	**X**
**Planning CT with and w/out i.v. contrast**	**X**						
**EORTC QoL brain Module and PAL**	**X**			**X**	**X**	**X**	**X**
**Documentation of medication**	**X**		**X**	**X**	**X**	**X**	**X**
**Documentation of AEs**	**X**		**X**	**X**	**(X)**	**(X)**	**(X)**
**Clinical, neurological examination**	**X**		**X**	**X**	**X**	**X**	**X**
**CRF Form**	**X**		**X**	**X**	**X**	**X**	**X**
